# Nationwide Geospatial Analysis to Identify Variations in Primary Cardiovascular Risk in Ethiopia

**DOI:** 10.1177/21501319241288312

**Published:** 2024-11-05

**Authors:** Yihun Mulugeta Alemu, Nasser Bagheri, Kinley Wangdi, Dan Chateau

**Affiliations:** 1 The Australian National University, Canberra, ACT, Australia; 2 Bahir Dar University, Bahir Dar, Ethiopia; 3 University of Canberra, Canberra, ACT, Australia

**Keywords:** cardiovascular risk prediction, WHO STEPS data, geospatial analysis, climate history data, geographical variations

## Abstract

**Background:**

Cardiovascular disease (CVD) varies across regions due to socioeconomic, cultural, lifestyle, healthcare access, and environmental factors.

**Objective:**

To find geographical variations in 10-year primary CVD risk and assess the impact of contextual factors on CVD risk.

**Method:**

Data from 2658 Ethiopians aged 40 to 69 years with no previous CVD who participated in a nationally representative World Health Organization (WHO) STEPS survey in 2015 were included in the analysis. The mean 10-year CVD risk for 450 enumeration areas (EA) was used to identify spatial autocorrelation (using Global Moran’s *I*) and CVD hot spots (using getas-Ord Gi*). Geographically Weighted Regression (GWR) analysis quantified the relationship between mean 10-year CVD risk and climate-related factors across areas.

**Result:**

The spatial autocorrelation analysis identified significant spatial variation in the 10-year CVD risk at the EA level, with a global Moran’s *I* value of 0.016. Statistically significant hot spot areas with 10-year CVD risk were identified in Addis Ababa (the capital), Benishangul Gumuz, SNNPR (Southern Nations, Nationalities, and Peoples’ Region), Amhara, Afar, Oromia, and Hareri regions. In a multivariable GWR analysis, average water vapor pressure was a statistically significant explanatory variable for the geographical variations in 10-year CVD risk.

**Conclusion:**

Hot spot areas for 10-year CVD risk were identified across numerous country regions rather than concentrated in a specific region. Alongside these hot spot areas, regions with a higher annual water vapor pressure (humidity) were identified as geographical targets for CVD prevention.

## Introduction

More than half a billion people around the world have been affected by cardiovascular disease (CVD), with a total of 20.5 million deaths in 2021.^1^ The majority (up to 80%) of heart attacks and strokes can be prevented.^2^ Advances in cardiovascular medicine in the last 50 years have brought tools and knowledge to mitigate harm to cardiovascular health.^3^ However, tools for diagnosing, preventing, and treating CVD are not reaching the segments of the world population that may need them most, highlighting the ongoing need to address health inequalities.^4^

Non-communicable diseases (NCDs) are the second leading cause of death in Sub-Saharan Africa (SSA), accounting for 35% of deaths. CVDs constitute 13% of all deaths and 37% of NCD deaths.^5^ From 1990 to 2019, CVD cases in 46 SSA countries increased by 131.7%, with age-standardized prevalence rates rising by 2.1%.^6^ In Nigeria, the pooled crude incidence of stroke was 26 per 100 000 person-years.^7^ In Ghana, the pooled prevalence of CVD in the general population was 10.3%.^8^ Similarly, in South Africa, the national prevalence of stroke was 1.3%, while the prevalence of coronary heart disease (CHD) was 4.3%.^9^ In Kenya, CVD accounted for over 10% of total deaths and 4% of total Disability-Adjusted Life Years (DALYs).^10^ In Ethiopia, the pooled prevalence of CVD was 5%, with 8% among hospital visitors and 2% in the general population.^11^

Ethiopia’s climate varies from humid rainforests to arid lowlands and cooler highlands. Since 1960, temperatures have risen by 1°C and precipitation has dropped by 20%, particularly in the south-central region, leading to increased rainfall variability and droughts. By the 2050s, temperatures may rise by 1.8°C and rainfall could decline by 20% in southern and central areas. Northern regions will face severe droughts and water stress. Climate risks are exacerbated by poverty and reliance on agriculture, potentially reducing Gross Domestic Product (GDP) by up to 10% by 2045, with urbanization adding strain on infrastructure and ongoing environmental degradation.^12^ Global climate changes continue to make Africa highly vulnerable, with extreme weather events affecting 19 million people and causing over 4000 deaths since early 2022.^13^

Social determinants of health, including socioeconomic, environmental, and psychosocial factors significantly contribute to variations in CVD risks.^14-16^ The social determinants of health, such as marital and occupational status, significantly influence CVD risk, morbidity, and mortality.^14^ For instance, marital status, such as being widowed, divorced, or separated, can influence CVD risk through variations in social support, mental stress, and economic stability, with higher stress and reduced support potentially increasing CVD risk.^17-19^ In addition, unemployment and climate factors like temperature and humidity affect CVD risk. Job insecurity and lower social status from unemployment contribute to higher CVD risk behaviors, highlighting the role of income and social protection.^20^ High humidity and temperature strain the cardiovascular system. Summer humidity was notably linked to higher hospitalizations for CVD, CHD, and stroke.^21-23^ Conversely, prolonged exposure to sunlight was associated with a lower CVD risk.^24-26^

CVD risk prediction equations help identify high-risk individuals and guide targeted treatments. The equations also raise population awareness and communicate risk knowledge to individuals, and communities, with applicability extending to low- and middle-income countries.^27-29^ The updated (2019) version of the World Health Organization (WHO) CVD risk prediction equation was designed to be easily recalibrated for many global regions using routinely available data. The laboratory-based version of the WHO 2019 CVD risk prediction equation estimates 10-year CVD risk for fatal and non-fatal CVD using age, smoking status, systolic blood pressure, history of diabetes, and total cholesterol.^30^ Even though social determinants of health are associated with CVD risk and the social determinants of health index is a tool or measure that effectively points out areas or regions with high CVD burden, improving CVD risk predictions, they are often excluded from models like the 2019 WHO CVD risk equation to ensure ease of application in Low- and Middle-Income Countries (LMICs).^31,32^ Geospatial analysis of 10-year CVD risk is essential for understanding spatial relationships, optimizing resource allocation, assessing and mitigating risks, identifying environmental impacts, supporting response efforts, and informing decision-making. Previous studies have identified spatial variations in CVD risk factors and events across regions like the USA, China, and South Africa, predominantly with participants from high- and middle-income countries, focusing on traditional risk factors and events.^33-38^ No published studies have identified geographical variations in 10-year CVD risk in Ethiopia; this study aims to fill this gap by quantifying the impact of contextual factors on CVD risk.

## Method

### Data Sources

This study used data from the WHO STEPS nationally representative survey of Ethiopian adults between 15 and 69 years of age, conducted from April to June 2015. Multiple sampling methods were used to identify potential participants: stratified, 3-stage cluster sampling, random sampling, and the Kish method (Appendix 1).^39^

For this analysis, we included participants aged 40 years and above from the STEPS survey. Participants who were pregnant or had a previous history of cardiovascular events (heart attack, angina, or stroke), and people with missing values on the input variables needed to calculate primary CVD risk were excluded. The proportion of the sample missing values on at least one of these input variables was 8.2% (total cholesterol of 6.9%, and systolic blood pressure of 1.3%). In addition, community-level WorldSIM satellite data^40^ on average 30 years (1970-2000) of annual temperature, and water vapor pressure (relative humidity) were linked to the survey data at the EA level to assess the potential impact of environmental factors on CVD risk within communities

## Study Variable

### Outcome Variable

Ten-year primary CVD risk was the main outcome, estimated by applying the laboratory-based sex-stratified WHO-2019 CVD risk equation.^41^ Age, smoking status (current vs non-current), systolic blood pressure (mmHg), history of diabetes (yes/no), and total cholesterol (mmol/L), measured in the WHO STEPS survey, were used to calculate the 10-year CVD. The risk equation has been recalibrated for use in the Ethiopian population using age- and sex-specific CVD incidence data from the 2017 update of the Global Burden of Disease Study (GBD 2017),^42^ and risk factor values from the Non-Communicable Diseases Risk Factor Collaboration (NCD-Risks).^43-45^ The WHO CVD risk prediction model includes interaction terms between age and each of the explanatory variables (smoking status, systolic blood pressure, history of diabetes, and total cholesterol).

### Explanatory Variables

Explanatory variables included in the spatial analysis were derived from the baseline survey data. These variables included: marriage status (never married, currently married, cohabiting, separated, divorced, widowed), and occupation (private skilled workers, farmers, traders, students, homemakers, or housewives, retired, unemployed but able to work, unemployed and unable-to-work). From these variables, we calculated proportions at the EA level, including the proportion of widowed, divorced, or separated individuals; the proportion of retired, unemployed, and those unable to work; and the proportion of individuals with low physical activity levels. We also calculated EA-level averages for humidity and temperature. These explanatory variables were included in the Geographically Weighted Regression (GWR) model. The proportion of the variable means the proportion of a certain variable per EA, for example, the proportion of retired/unable-to-work per EA. Physical activity levels were measured using the Global Physical Activity Questionnaire (GPAQ).^46,47^ Details of the definition and coding of the variables are presented in Appendix 1. Average annual temperature and water vapor pressure data for the 30 years (1970-2000) were calculated from WorldSIM’s 1 km spatial resolution climate surfaces for EA level. These datasets consist of spatially interpolated monthly climate data for each year during the specified time frame.^40^ Initially, we obtained geospatial raster data for each EA and subsequently transformed it into numerical features. We included social determinants of health, such as marital and occupational status, as explanatory variables. Marital status, including proportions of widowed, divorced, or separated individuals, can influence health equity and geospatial variations of CVD risk through factors like income, social protection, and social inclusion. Occupational status, including proportions of retired, unemployed, and those unable to work, impacts health inequity and CVD risk variations due to challenges related to unemployment and job insecurity.^48^ Long-term humidity and temperature (1970-2000) were included as explanatory variables due to their effect on CVD risk. Temperature extremes and high humidity can impair the cardiovascular system, leading to increased risks of heart attacks, strokes, and CVD-related hospital admissions.^49^ On the other hand, sun exposure reduces hypertension risk and CVD deaths.^50^ Lower sunlight exposure was also linked to lower HDL levels.^51^ The link between outdoor light exposure and heart failure suggests that moderate light exposure may prevent heart failure.^26^

### Data Analysis

We first calculated the 10-year CVD risk for each participant using the WHO-2019 laboratory-based CVD risk equation. Individual level scores were weighted using survey weights provided (based on age, sex, and area of residence) to ensure that scores derived from a data set are representative of those in the population.^52^ Single imputation was used to impute missing values for the explanatory variables among eligible participants, excluding factors required for estimating CVD risk, such as marital status. Numeric variables with missing values were substituted with the computed median for that particular variable. In the case of categorical variables, missing values were replaced with the mode, representing the most frequent category.^53^ The proportion of data missing for each of the explanatory variables was less than 1%.

**Figure 1.**
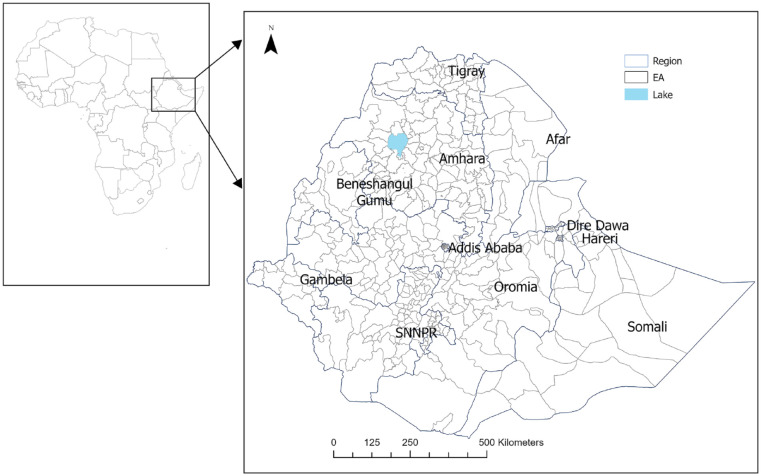
The location of Ethiopia, regional states, and enumeration areas.

**Figure 2.**
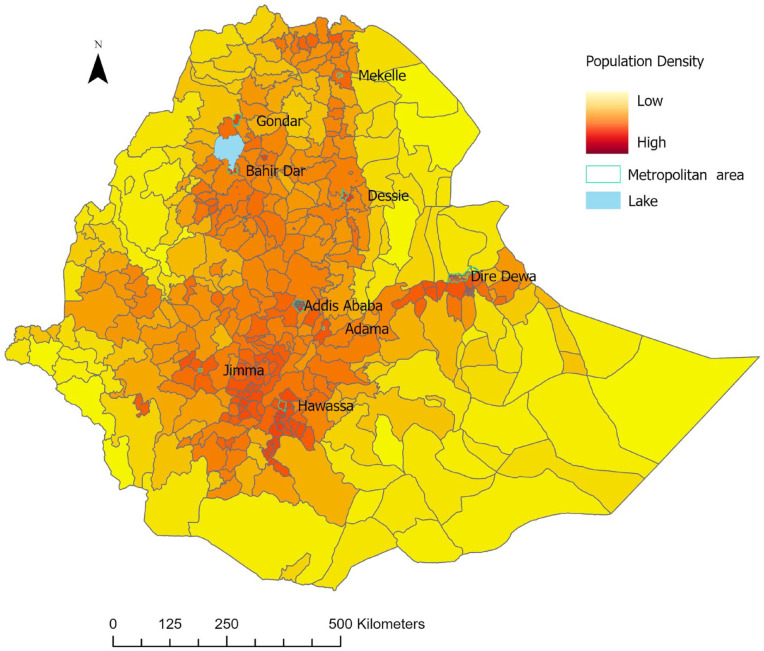
A map layer of 9 metropolitan areas in Ethiopia and population density.

Ten-year CVD risk was calculated individually and aggregated at the EA level by determining the mean CVD risk. The mean/proportions of explanatory variables within an EA were computed similarly. Stata version 17 statistical analysis software (Stata Corp. Stata Statistical Software: Release 17. College Station, TX: Stata Corp LP. 2023) was used to compute the 10-year CVD risk and other descriptive statistics. Our data set consisted of 450 EA, while the Ethiopia level 3 geographical administrative districts are 684 (Figure 1).^54^ EAs that were part of the survey were merged with near or adjacent districts to enable complete coverage of Ethiopia. The missing regions should not alter the results substantially because these regions have a low proportion of individuals over age 39 years. We included a map layer showing 9 metropolitan areas in Ethiopia and the country’s population density (Figure 2) to examine CVD risk patterns in major cities and with population density. We used the High-Resolution Population Density Maps and Demographic Estimates of the general population in 2022 for Ethiopia to calculate the population density of specific areas.^55^ District-level areas were used as catchment areas. We summed the high-resolution population values of each district to obtain the total population and then divided this total by the area of the district to estimate the population density. R (version 4.3.0) was used to construct a forest plot. ArcGIS Pro 3. x 2023 (Esri, USA) software was used for spatial analyses and for retrieving geospatial climate history data (average annual—temperature and—water vapor pressure) which were linked to the WHO STEPS survey data sets.

### Spatial Analysis

We employed 3 distinct spatial methods—(1) Spatial Autocorrelation: using Global Moran’s, (2) Hot spots analysis: using Getas-Ord Gi*, and (3) Geographically Weighted Regression was used to determine whether the relationship between variables remained stationary or varied across the study areas—each serving a specific purpose and complementing one another in the geospatial analysis of 10-year CVD risk. These approaches are highly applicable for informing public health interventions and guiding resource allocations.^56-68^

Global Moran’s *I* tool assesses spatial autocorrelation by considering both the locations and values of features simultaneously.^56,57^ Global Moran’s *I* help in understanding the degree of similarity between neighboring regions regarding CVD risk. Global Moran’s *I* was done to assess whether there was spatial clustering (similarity), dispersion (dissimilarity), or random distribution of the 10-year CVD risks across the entire study area.^58,59^ Moran’s, *I* value close to −1 would indicate that the 10-year CVD risk was dispersed, while Moran’s *I* value close to + 1 would indicate the 10-year CVD risk was clustered. A Moran’s *I* value of 0 indicates the 10-year CVD risk was randomly distributed.^60,61^

The Getas-Ord Gi* statistics were used to identify potential clusters in the spatial pattern of average 10-year CVD risk at the EAs or district levels.^62^ We employed inverse distance and inverse distance square weighting techniques to compute the Getas-Ord Gi*, and both had similar results. The Getas-Ord Gi* statistic was a *Z*-score that identifies areas of higher or lower values by comparing them to a normal probability distribution and provides a measure of the local concentration of increased 10-year CVD risks (clustering of areas with higher values of mean CVD risk score). The *Z*-score and *P*-value determined the statistically significant difference in clustering. High Gi* indicates a “hot spot” of 10-year CVD risk (area with a high mean risk) while low GI* indicates a “cold spot” (area with a low mean risk).^56^ Gaisford* hot spot analysis technique helps determine areas where the CVD risk was concentrated, facilitating targeted interventions and/or strategies.^62^

### Spatial Regression

GWR allows the relationships between independent and dependent variables to vary over the space.^65,66^ GWR was used to evaluate a local model of an explanatory variable to predict the outcome variable by fitting a regression equation at each data point. GWR allows for examining how the relationship between explanatory variables and CVD risk varies spatially. GWR considers the assessment of spatial heterogeneity by estimating local regression parameters that vary across geographic space, enabling the spatial variability that Ordinary Least Square Regression (OLS) might overlook.^67,68^ We initially computed multivariable OLS for 7 explanatory variables (Appendix 1)^63,64^ and estimated the residuals of the OLS model for Moran’s Autocorrelation. We observed statistically significant autocorrelation. Then, we computed GWR regression for the 7 explanatory variables (mean age; proportion of—males,—widowed/divorced/separated individuals,—retired/unemployed and unable-to-work,—low physical activity; and average water—vapor pressure, and—temperature). The GWR coefficients were calculated and highlighted where the relationship between CVD risk and the covariate was relatively stronger or weaker across the communities in Ethiopia. The statistical significance of each explanatory variable in the GWR model was examined using the table output. A GWR significance value of 1 indicates a statistically significant parameter estimate at the 95% confidence interval (CI) within a specific EA, while a value of 0 signifies non-significance for that EA.^62^ We computed *R*^2^, adjusted *R*^2^, and Akaike’s Information Criterion for model diagnostics of GWR (Appendix 1).

### Ethical Approval

The study obtained ethics approval from Bahir Dar University, College of Medicine and Health Science College Research Ethics Committee, Ethiopia with protocol number 166/2021, and the Australian National University Human Ethics Committee, Australia with protocol number 2021/436.

## Results

### Mean Overall Estimated 10-Year CVD Risk in Ethiopia

**Figure 3.**
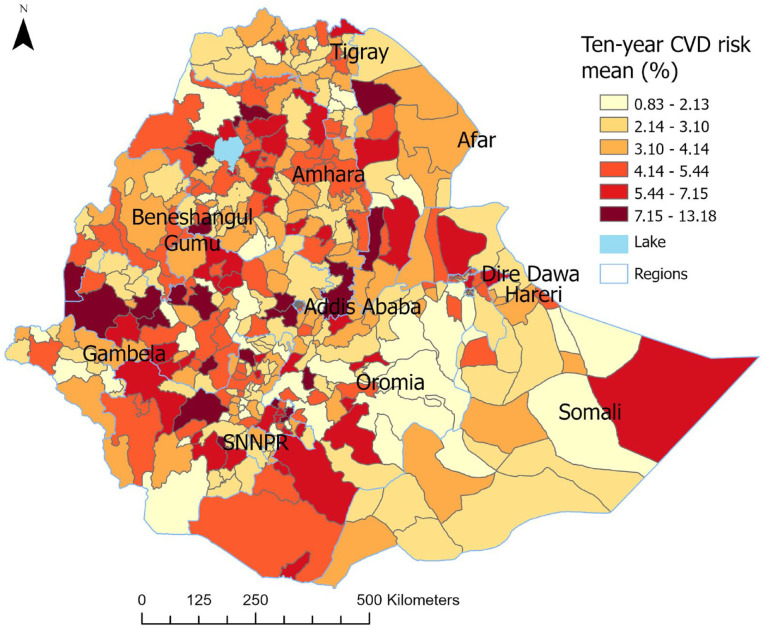
A mean 10-year CVD risk in 450 enumeration areas across Ethiopia using the WHO 2015 STEP survey.

The observed mean 10-year CVD risk was highest in the capital (Addis Ababa; 5%) followed by the Somali regional state (4.8%). The lowest regional mean 10-year CVD risk was in the Tigray region (3.4%; Table 1). The average 10-year CVD risk in EAs varies across different districts of the country, ranging from 0.83% to 13.18% (Figure 3). The forest plot illustrates the overall estimated 10-year CVD risk and the corresponding confidence intervals within the 450 EAs (Appendix 1-Figure A2). In Ethiopia, major cities with metropolitan populations include Addis Ababa, the capital and largest city; Gondar; Mekelle; Adama; Awasa; Bahir Dar; Dire Dawa; Dessie; and Jimma.^69^ In subgroup analysis, the observed mean 10-year CVD risk was 5.3% in metropolitan areas compared to 3.8% in non-metropolitan areas.

**Table 1.** Number of Populations, Number of Enumeration Areas, Study Participants, and Mean 10-year CVD Risk by Region Across Ethiopia Using the WHO 2015 STEP Survey.

	Regional sates	Number of enumeration areas	Study participants	Mean 10-year CVD risk %
1	Tigray	33	315	3.43
2	Afar	17	66	3.56
3	Amhara	92	573	3.72
4	Oromia	107	577	4.34
5	Somali	31	246	4.80
6	Benishangul Gumuz	19	90	4.05
7	SNNPR	75	401	4.17
8	Gambela	12	43	4.13
9	Hareri	11	57	4.03
10	Addis Ababa	14	67	5.09
11	Dire Dawa	39	223	3.82
Total		450	2658	

Abbreviation: SNNPR, Southern Nations, Nationalities, and Peoples’ Region.

**Figure 4.**
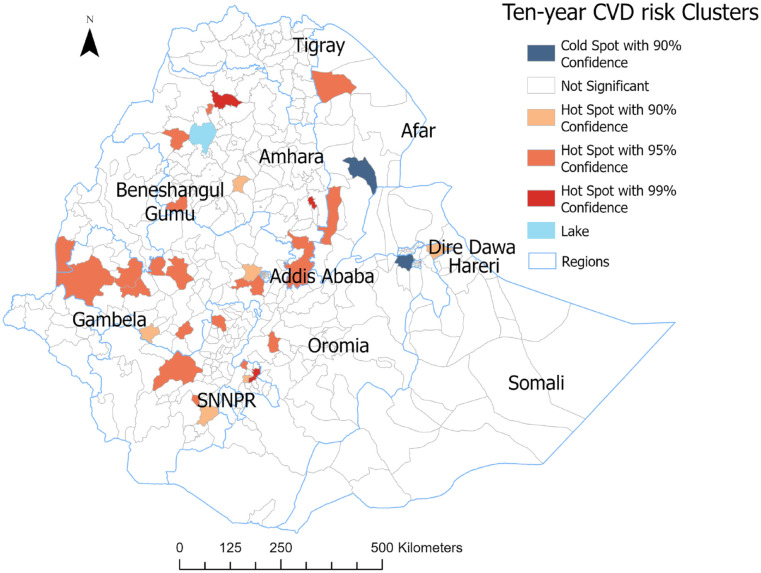
Hot spot and cold spot of mean 10-year CVD risk in Ethiopia using the WHO 2015 STEP survey.

### Spatial Autocorrelation and Hot Spot of 10-Year Mean CVD Risk

The spatial autocorrelation analysis identified significant spatial variation of 10-year CVD risk across Ethiopia with a global Moran’s *I* value of 0.016 (*P* < .05; Appendix 1-Figure A1). Thirty-four hot spot areas were identified, indicating a higher 10-year CVD risk (Figure 4). Three hot spot areas of 10-year CVD risk were at 99% CI, 23 at 95% CI, and 8 at 90% CI. These hot spots were in Addis Ababa (the capital), Benishangul Gumuz, SNNPR (Southern Nations, Nationalities, and Peoples’ Region), Amhara, Afar, Oromia, and Hareri regions. “Cold spots” with 10-year CVD risk were identified in Afar and Oromia regions (90% CI level).

### Geographically Weighted Regression

**Figure 5.**
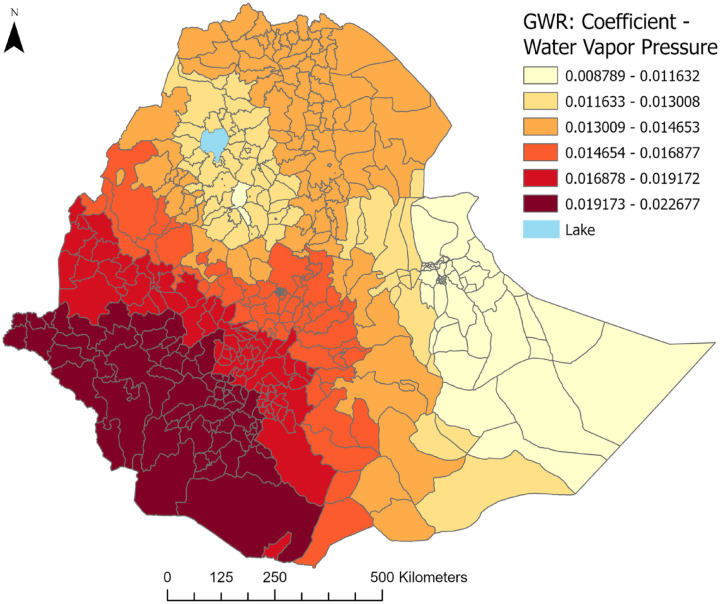
Variations in Ethiopia’s mean 10-year CVD risk are driven by average annual water pressure (1970-2000) using the WHO 2015 STEP survey.

After conducting a multivariable GWR model analysis involving 7 explanatory variables—mean age, proportion of males, widowed/divorced/separated individuals, retired/unable-to-work individuals, low physical activity level, average vapor pressure/relative humidity, and temperature—the results showed a positive association between mean 10-year CVD risk and the average 30-year water vapor pressure (humidity), particularly pronounced in Western and Southwestern Ethiopia (Figure 5). The model-adjusted *R*^2^ was .59 (Appendix 1-Tables A1-A3).

## Discussion

The main findings from this study identified a significant spatial correlation in the 10-year mean CVD risk across Ethiopia. Hot spot areas showed higher 10-year average CVD risk, which was distributed throughout the nation rather than concentrated in a particular area. A significant factor in spatial variations of the 10-year CVD risk was the average relative humidity/water vapor pressure.

In our study, the 10-year CVD risk was higher in metropolitan areas compared to non-metropolitan areas. Similarly, studies from several African countries showed a higher prevalence of high blood pressure, obesity, and diabetes in metropolitan cities than in rural areas. This disparity could be associated with lifestyle differences between these regions.^70^ In metropolitan areas, sedentary lifestyles, access to processed foods, and higher rates of smoking and excess alcohol consumption contribute to higher CVD risk.^71^ In addition to lifestyle factors, higher air pollution and rapid urbanization in metropolitan areas could also increase CVD risk compared to non-metropolitan areas.^72^

Spatial analyses identified significant spatial autocorrelation of 10-year CVD risk in Ethiopia. This study’s findings showed patterns of 10-year CVD risk clustering and hot spots varied across the country. Higher 10-year CVD risk in certain geographical locations and its surroundings or hot spots could be due to the clustering of CVD risk behaviors such as smoking, low-physical activities, or consumption of unhealthy diet^73-75^; accumulation of high-risk groups in nearby geographical units^76^; and/or disparities in the health systems across communities.^77^ In addition, significant geographical variation in 10-year CVD risk also highlights differences in age distribution, gender ratio, and socioeconomic status between areas. Environmental aspects such as pollution levels, availability of green spaces, the walkability of areas, and climatic variations could also influence CVD risk variations across areas.^78,79^ Understanding the influence of these factors on area-level disparities in CVD risk enables healthcare professionals and policymakers to adapt interventions and strategies aimed at mitigating CVD risks at regional levels. Targeted efforts, such as intensified CVD screenings and therapeutic measures, should be prioritized in hot spot areas with higher 10-year CVD risk.^80-83^ Hot spot areas in our study were major cities and surrounding regions such as Addis Ababa (the capital), Gondar, Jimma, Harar, near Awasa, and near Mekelle; these CVD hot spots often include high-population density urban areas where factors such as air pollution, sedentary lifestyles, and unhealthy diets contribute to higher CVD risk were more common compared to rural counterparts.^72,79^ Additionally, Hot spots were identified in the Benishangul-Gumuz region, where economic and social factors, environmental conditions, and limited access to healthcare further increase the risk of CVD in this more disadvantaged area.^16,84^

Published literature described the effects of meteorological parameters on CVD outcomes. Changes in temperature and water-vapor pressure were associated with increased blood pressure, lipid levels, inflammatory markers, plasma cholesterol, plasma fibrinogen, and heart rate variability in the elderly or persons with preexisting CVD.^85^ In addition, another study also identified that long-term exposure to higher average humidity and its variability showed positive associations with CVD hospitalizations.^21^ In our study, a positive association was found between the average annual water vapor pressure (1970-2000) and the 10-year CVD risk, particularly evident in the Western and Southwestern regions of the country, as indicated by higher GWR coefficients. This could also be due to higher humidity levels potentially worsening respiratory and cardiovascular conditions in vulnerable individuals.^15^ Geographical features, such as low-lying areas or regions prone to flooding, may exacerbate the impact of increased water vapor pressure on cardiovascular health by leading to higher humidity levels, potentially contributing to cardiovascular risk factors like mold growth and air pollution.^86,87^

Humidity was often a key weather factor influencing overall mortality and specific causes like CVD and stroke.^88^ Climate change is causing the Earth to become hotter and more humid, significantly increasing the risk of mortality by affecting cardiovascular and respiratory health.^22^ Humidity, a key weather factor affecting mortality and specific causes like CVD and stroke, is rising due to climate change, significantly increasing cardiovascular and respiratory health risks.^22,88^ Rising global temperatures increase humidity, exacerbating heat stress, impedes sweating, and raises heart rate and blood pressure, worsening CVD risk.^21,89-91^

Ethiopia has faced worsening climate extremes over recent decades, including severe droughts, floods, and a rise in mean annual temperature, with trends expected to accelerate.^92-94^ Climate change in Ethiopia is exacerbating health challenges, increasing mortality and morbidity from floods, heat waves, and respiratory illnesses. Our study found higher humidity associated with increased CVD risk, emphasizing the need for targeted health interventions in climate adaptation.^95,96^

### Strengths and Limitations of the Study

The strength of this study is the use of nationally representative datasets, which enhance the credibility of the evidence, ensure diversity, and statistical power, and facilitate generalizability of findings, thus informing policy decisions with increased trustworthiness.^97,98^ The study used comprehensive geospatial analysis, revealing CVD risk patterns, identifying vulnerable populations, and guiding targeted interventions and resource allocation in different regions of Ethiopia.^99,100^ This study’s linkage of a 30-year climate history dataset, including water vapor pressure data, offers a perspective for investigating environmental factors linked to CVD risk, enabling more comprehensive geospatial analyses and enhancing understanding of climate-health interactions for improved targeted interventions.^101^ While providing valuable insights into CVD risk in Ethiopia, this study’s aggregation of mean CVD risk at the district or EA level may obscure important individual variations.^102^ This study used WHO STEPS data from 2015 and climate data from 1970 to 2000. The 2015 data may not fully capture changes in the last 9 years. Climate factors influencing CVD risk, such as water vapor pressure and temperature, require prolonged exposure to reveal long-term and lagged effects. The chosen period helps understand the delayed physiological impacts of climate on CVD risk factors in populations without previous CVD; however, it doesn’t reflect changes from 2001 to 2015 or the last 9 years.^103^

## Conclusion

Hot spot areas for 10-year CVD risk were identified across numerous country regions, rather than concentrated in a specific region. Alongside these hot spot areas, regions with a higher annual water vapor pressure (humidity) were identified as geographical targets for CVD prevention.

## Figures and Tables

**Figure A1.**
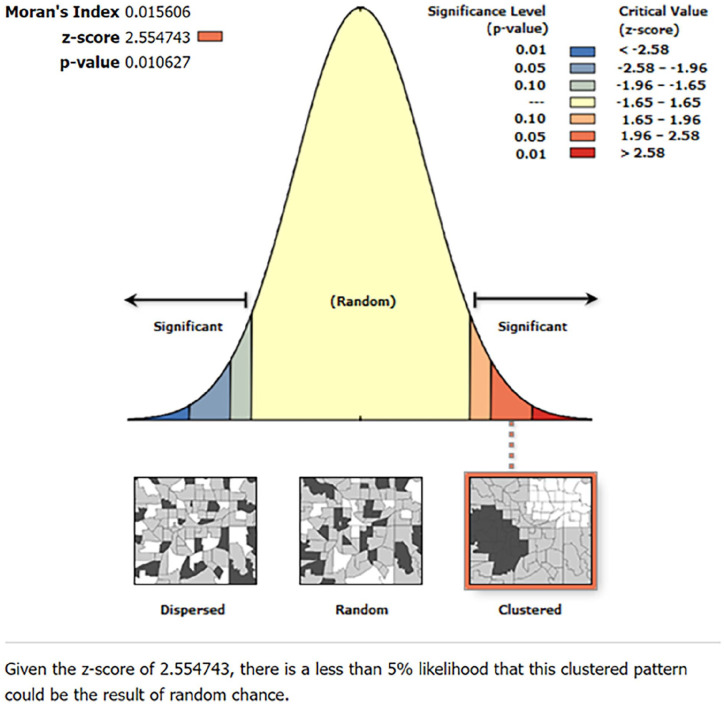
Moran’s Index of mean 10-year CVD risk in 450 enumeration areas across Ethiopia using the WHO 2015 STEP survey.

**Figure A2.**
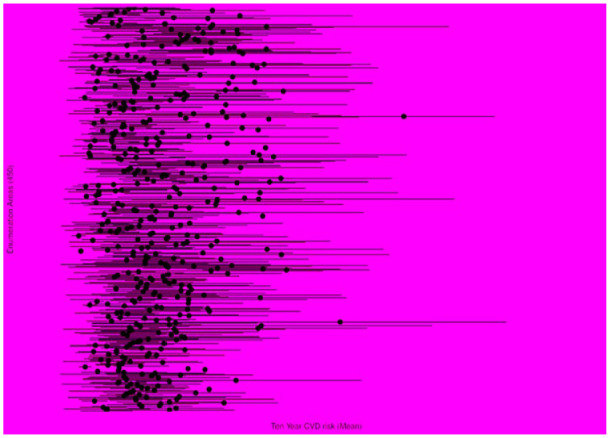
Forest plot for mean 10-year CVD risk estimates and 95%CI confidence intervals in 450 enumeration areas.

## Appendix 1

### Nationwide Geospatial Analysis to Identify Variations in Primary Cardiovascular Risk in Ethiopia

#### Data sources

All 11 regions in the country were included, each divided into administrative zones. The administrative zones were divided into districts (“Woreda”). The districts were also further divided into “Kebele.” The kebeles are the smallest administrative units in Ethiopia. EAs were the principal sampling units for the survey. The EAs comprise on average 100 households in urban and rural settings; on average a total of 20 households per EA were selected using systematic random sampling. One respondent per household was included using the Kish method.^1^

### Definitions of Terms

#### Explanatory variables ascertained from the WHO STEPS Ethiopia survey

##### Exercise level

According to WHO criteria, the Global Physical Activity Questionnaire (GPAQ) measured physical activity. Three domains measured physical activities: work, transport, and recreation-related activities.^2,3^ We calculated MET (metabolic equivalent) for 3 domains. Work: moderate MET value = 4.0, and vigorous MET value = 8.0. Transport, cycling, and walking: moderate MET value = 4.0. Recreation: moderate MET value = 4.0, and vigorous MET value = 8.0. High-level physical activity was when a person engaged in vigorous-intensity physical activity for at least 3 days (a minimum of 1500 MET-min) per week or 7 days of any combination of moderate- or vigorous-intensity activities in a week, achieving a minimum of 3000 MET per week. Moderate-level physical activity was defined as a person who exercised 3 or more days with vigorous-intensity activity for at least 20 min per day, or walking for at least 30 min per day for 5 or more days of any combination of walking, moderate- or vigorous-intensity activities, and achieving a minimum of at least 600 MET-min per week. Low-level physical activity occurs when a person does not meet the criteria mentioned earlier.^2,3^

**Table A1.** Ordinary Least Square Result Summary of Mean 10-year CVD Risk.

Variable	Coefficient	Robust SE	Robust *t*	Robust Pr	VIF
Intercept	−.11	0.008	−12.97	<0.001*	−
Proportion of male	.009	0.002	4.12	<0.001*	1.27
Mean age	.002	0.0001	19.02	<0.001*	1.67
Proportion of widowed/divorced/separated	.01	0.008	1.82	0.06	1.22
Proportion of retired/unemployed and unable to work	.02	0.009	3.15	<0.001*	1.09
The proportion of low physical activity within the community	.01	0.0006	1.72	0.08	1.10
Average water vapor pressure	.01	0.004	3.62	<0.001*	5.24
Average temperature	−.001	0.0003	−2.81	0.005*	5.16

* =significant at *p*-value < 0.05.

**Table A2.** OLS Model Diagnostics of Mean 10-year CVD Risk.

Number of observations	450	Akaike’s information criterion (AICc)	−2686.79
Multiple *R*^2^	.57	Adjusted *R*^2^	.56

**Table A3.** GWR Model Diagnostics of Mean 10-year CVD Risk.

Number of observations	450	Akaike’s information criterion (AICc)	−2737.46
Multiple *R*^2^	.61	Adjusted *R*^2^	.59

### References

1. Ethiopia Public Health Institute (2016) Ethiopia steps report on risk factors for non-communicable diseases and prevalence of selected NCDS: 2016, Addis Abab Ethiopia.

2. World Health Organization (2010) Guidelines Global Recommendations on Physical Activity for Health, WHO, 2010.

3. World Health Organization (2010) GPAQ (Global Physical Activity Questionnaire), WHO 2010.
